# ﻿Morphological and phylogenetic analyses reveal three new species associated with *Pueraria* from Xizang Autonomous Region, China

**DOI:** 10.3897/mycokeys.125.174645

**Published:** 2025-11-27

**Authors:** Gui-Li Zhao, Yong-Zhong Lu, Xing-Juan Xiao, Ying Liu, Qiang Chen, Ning-Guo Liu

**Affiliations:** 1 School of Chemical Engineering, Guizhou Institute of Technology, Guiyang 550025, China Guizhou Key Laboratory of Agricultural Microbiology, Guizhou Academy of Agricultural Sciences Guiyang China; 2 Guizhou Key Laboratory of Agricultural Microbiology, Guizhou Academy of Agricultural Sciences, Guiyang 550009, China School of Chemical Engineering, Guizhou Institute of Technology Guiyang China; 3 School of Food and Pharmaceutical Engineering, Guizhou Institute of Technology, Guiyang 550025, China School of Food and Pharmaceutical Engineering, Guizhou Institute of Technology Guiyang China

**Keywords:** 3 new taxa, Dothideomycetes, phylogeny, *Pueraria*-associated microfungi, taxonomy

## Abstract

During a survey of microfungi associated with *Pueraria* in the Xizang Autonomous Region, China, three saprobic taxa were isolated from the dead vines of *Pueraria* sp. Based on morphological characteristics and multi-gene phylogenetic analyses, the three taxa are identified as new species, viz., *Hermatomyces nyingchiensis*, *H. xizangensis*, and *Rhytidhysteron xizangense*. Morphological descriptions and illustrations of these new collections, along with a synopsis of *Hermatomyces* species, are provided.

## ﻿Introduction

Species of *Pueraria* (Leguminosae) are widely distributed across Asia, North America, and South America (Wang et al. 2020). They exhibit strong colonization ability and ecological adaptability, enabling them to survive and thrive even in post-mining environments, thereby contributing to the restoration of degraded lands (Singhal 2009). Members of *Pueraria* contain numerous compounds, including puerarin, isoflavones, flavones, flavonols, coumestrols, and other related phytochemicals (Chen et al. 2018; Wang et al. 2020; Chen et al. 2021; Wang et al. 2021; Zhang et al. 2021). These compounds possess diverse pharmacological activities, including anti-alcoholism (Xiong et al. 2010), antioxidant (Zhang et al. 2017; Son et al. 2019), hepatoprotective (Xu et al. 2013), and antidiabetic effects (Shukla et al. 2017; Wang et al. 2017; Sun et al. 2019). Although previous studies have investigated the arbuscular mycorrhizal fungi associated with *Pueraria* (Guo et al. 2022), research on saprophytic fungi related to *Pueraria* remains limited. In this study, we aim to explore the diversity of fungi associated with *Pueraria*, with a focus on saprobic fungal communities.

*Hermatomyces* was established by Spegazzini (1910), with *H. tucumanensis* as the type species. It was initially placed within Lophiotremataceae (Doilom et al. 2016; Tibpromma et al. 2017). However, Hashimoto et al. (2017) excluded *Hermatomyces* from Lophiotremataceae and reinstated Hermatomycetaceae based on molecular evidence. Most species of *Hermatomyces* are known from their asexual morph, characterized by sporodochial conidiomata and dimorphic conidia (cylindrical and lenticular) (Doilom et al. 2016; Tibpromma et al. 2017; Koukol et al. 2018; Ren et al. 2021; Zhang et al. 2023; Liu et al. 2024). The sexual morph was recently described and is characterized by dark brown to black ascomata with a central ostiole, 8-spored, bitunicate asci, and hyaline, broadly fusiform, 1-septate ascospores (de Silva et al. 2022).

*Rhytidhysteron* was originally established by Spegazzini (1881) and was historically placed within Patellariaceae (Bezerra and Kimbrough 1982). However, molecular evidence provided by Boehm et al. (2009) demonstrated that *Rhytidhysteron* is not phylogenetically related to Patellariaceae but rather belongs to Hysteriaceae. Currently, 44 records of *Rhytidhysteron* are listed in Index Fungorum (http://www.indexfungorum.org, accessed 13 October 2025). Most species are known from their sexual morphs, whereas only four species have been recognized from their asexual morphs, which produce two types of conidia: aposphaeria-like and diplodia-like (Samuels and Müller 1979; Thambugala et al. 2016; Ren et al. 2022). The sexual morph of *Rhytidhysteron* is characterized by prominent, sizable, navicular ascomata, typically with perpendicular striations along the margins (Thambugala et al. 2016; de Silva et al. 2020; Wanasinghe et al. 2021; Ren et al. 2022; Du et al. 2023). The ascospores are pigmented, septate, and muriform to sub-muriform (Thambugala et al. 2016; Ren et al. 2022). Species within this genus play essential ecological roles, functioning as saprobes, endophytes, and mild pathogens on woody substrates in terrestrial and aquatic environments. Occasionally, some species have also been reported as human pathogens (Thambugala et al. 2016; Soto-Medina and Lücking 2017; de Silva et al. 2020; Wanasinghe et al. 2021; Ren et al. 2022; Du et al. 2023).

We aim to carry out a survey investigating fungal diversity associated with *Pueraria* sp. from the Xizang Autonomous Region, China. During this survey, three taxa were obtained. Based on morphological characterization, illustrations, and multi-gene phylogenetic analyses, *Hermatomyces nyingchiensis* sp. nov., *H. xizangensis* sp. nov., and *Rhytidhysteron xizangense* sp. nov. are described herein.

## ﻿Materials and methods

### ﻿Specimen collection, examination, and isolation

Specimens were collected from dead vines of *Pueraria* species in the Xizang Autonomous Region, China, and packed into plastic bags with recorded details such as collection date, locality, and host. The specimens were transported to the laboratory for examination and isolation. Morphological examination and single-spore isolations followed the methodologies described by Senanayake et al. (2020). Germinated spores were carefully transferred onto fresh potato dextrose agar (PDA) medium and incubated at room temperature. During this period, morphological characteristics of the colonies, such as color, form, and texture, were observed and recorded. Fungal colonies growing on the substrate were examined using Nikon SMZ 745 and SMZ 800N (Nikon, Tokyo, Japan) dissecting microscopes. Detailed microscopic observations and image capture were performed with a Nikon ECLIPSE microscope equipped with a Nikon DS-Ri2 digital camera. Pure cultures were deposited in the Guizhou Culture Collection (GZCC), Guiyang, China, and dried specimens were preserved in the Herbarium of Cryptogams, Kunming Institute of Botany, Academia Sinica (HAKS), Kunming, China. The new species were registered in Fungal Names (https://nmdc.cn/fungalnames/).

### ﻿DNA extraction, PCR amplification, and sequencing

Molecular analyses were conducted following the protocols outlined by Dissanayake et al. (2020). Genomic DNA was extracted from freshly grown mycelia on PDA plates using the Ezup Column Fungi Genomic DNA Purification Kit (Sangon Biotech, Shanghai, China), according to the manufacturer’s instructions. Amplification of the large subunit ribosomal RNA region (LSU) was performed with the primers LR0R and LR5 (Vilgalys and Hester 1990); the internal transcribed spacer (ITS) region with ITS5 and ITS4 (White et al. 1990); the translation elongation factor 1-alpha (*tef*1-α) with EF1-983F and EF1-2218R (Rehner and Buckley 2005); the RNA polymerase II second largest subunit (*rpb*2) with fRPB2-5F and fRPB2-7cR (Liu et al. 1999); the β-tubulin (*tub*2) with primers T1 and T22 (O’Donnell and Cigelnik 1997); and the small subunit ribosomal RNA region (SSU) with NS1 and NS4 (White et al. 1990). Polymerase chain reaction (PCR) was conducted in a 25 μl volume containing 21 μl of 1.1 × T3 Super PCR Mix (Qingke Biotech, Chongqing, China), 1 μl of each primer, and 2 μl of DNA template. PCR amplification was initiated by denaturation at 98 °C for 2 min, followed by 40 cycles of 98 °C for 10 s, annealing at 55 °C for 1 min, and extension at 72 °C for 30 s. A final extension was performed at 72 °C for 2 min. Purification and sequencing of the PCR products were conducted by Tsingke Biotechnology Co., Ltd. (Beijing, China).

### ﻿Phylogenetic analyses

All newly generated sequences were checked using BioEdit v. 7.0.5.3 to assess sequence quality. Forward and reverse sequences were assembled using SeqMan v. 7.0.0 (DNASTAR, Madison, WI, USA) (Clewley 1995). Sequences used in phylogenetic analyses were obtained from GenBank (http://blast.ncbi.nlm.nih.gov/) based on BLASTn search results and relevant literature (Doilom et al. 2016; Tibpromma et al. 2016, 2017; Soto-Medina and Lücking 2017; Koukol et al. 2018; de Silva et al. 2020; Ren et al. 2021; Du et al. 2023; Zhang et al. 2023). Multiple sequence alignments were carried out using the online MAFFT version 7 (https://mafft.cbrc.jp/alignment/server/) (Katoh and Standley 2013). Single alignment trimming was conducted using trimAl v. 1.2 (Capella-Gutiérrez et al. 2009) with the gappyout option. Maximum Likelihood (ML) and Bayesian Inference (BI) analyses were conducted online via the CIPRES Science Gateway platform (https://www.phylo.org/portal2/home.action). The ML trees were constructed using RAxML-HPC v. 8 on XSEDE (version 8.2.12), employing the GTRGAMMA model and 1,000 bootstrap replicates. Posterior probabilities (PP) were estimated through BI analyses using the Markov chain Monte Carlo (MCMC) algorithm implemented in MrBayes on XSEDE (version 3.2.7a) (Ronquist et al. 2012). In *Hermatomyces*, the optimal models applied were: TNe+R2 for LSU, TIM2e+I+G4 for ITS, TN+F+R3 for *tef*1-α, TNe+G4 for *rpb*2, and K2P+I for *tub*2. In *Rhytidhysteron*, the optimal models were: TN+F+R2 for LSU, K2P+G4 for ITS, K2P+I for SSU, and TIM2e+I+G4 for *tef*1-α. Markov chains were run for 50,000,000 generations, with trees sampled every 100 generations. Phylogenetic trees were visualized using FigTree v. 1.4.4, and final figure layouts were edited using Adobe Illustrator CC 2019 (version 23.1.0; Adobe Systems, San Jose, CA, USA). The sequences generated in this study were submitted to GenBank, and their accession numbers are listed in Tables 1, 2.

**Table 1.** Strains and sequence accession numbers included for analysis of *Hermatomyces*. The newly generated sequences are in bold. “^T^” indicates the type strains. NA indicates sequence unavailability.

Fungal Species	Strain numbers	Conidia type	Country	LSU	ITS	*tef*1-α	*rpb*2	*tub*2	References
*Anteaglonium parvulum*	MFLUCC 14-0823	-	Thailand	KU922917	NA	KU922922	NA	NA	Jayasiri et al. (2016)
*A. thailandicum*	MFLUCC 14-0816	-	Thailand	KU922909	NA	KU922920	NA	NA	Jayasiri et al. (2016)
*Hermatomyces amphisporus*	CBS 146613	2	USA	LR812662	LR812662	LR812657	LR812668	LR812673	Delgado et al. (2020)
*H. amphisporus*	CBS 146614	2	USA	LR812666	LR812666	LR812660	LR812671	LR812676	Delgado et al. (2020)
*H. anomianthi*	MFLUCC 21-0202^T^	1 (Sexual)	Thailand	OK655817	OL413437	OM117546	NA	NA	de Silva et al. (2022)
*H. bauhiniae*	MFLUCC 16-0395^T^	2	Thailand	MK443378	MK443382	MK443384	MK443386	NA	Hyde et al. (2019)
*H. bifurcatus*	CCF 5899	2	Panama	NA	LS398262	LS398416	LS398343	LS398441	Koukol et al. (2018)
*H. bifurcatus*	CCF 5900^T^	2	Panama	NA	LS398263	LS398417	LS398344	LS398442	Koukol et al. (2018)
*H. clematidis*	MFLUCC 17-2085^T^	2	Thailand	MT214556	MT310603	MT394735	MT394684	NA	Phukhamsakda et al. (2020)
*H. constrictus*	CCF 5904^T^	2	Panama	LS398264	LS398264	LS398418	LS398345	NA	Koukol et al. (2018)
*H. griseomarginatus*	PRC 7201	1	Benin	NA	PP779919	PP840395	PP840388	NA	Koukol et al. (2025)
*H. griseomarginatus*	PRC 7203^T^	1	Benin	NA	PP779918	PP840396	PP840389	NA	Koukol et al. (2025)
*H. hainanensis*	GZCC 23-0592^T^	2	China	OR091329	OR098708	NA	NA	NA	Zhang et al. (2023)
*H. hongheensis*	KUNCC 23-13503^T^	1	China	PP189897	PQ340461	PQ456948	PQ348601	NA	Shen et al. (2024)
*H. hongheensis*	KUNCC 23-14231^T^	1	China	PP189898	PQ340462	PQ456949	NA	NA	Shen et al. (2024)
*H. indicus* (*H. thailandicus*)	MFLUCC 14-1145	2	Thailand	KU764694	KU144922	KU872756	KU712490	NA	Doilom et al. (2016)
*H. indicus* (*H. thailandicus*)	MFLUCC 14-1143^T^	2	Thailand	KU764692	KU144920	KU872754	KU712488	NA	Doilom et al. (2016)
*H. iriomotensis*	KH 361^T^	2	Japan	LC194367	LC194483	LC194394	LC194449	NA	Hashimoto et al. (2017)
*H. jinghaensis*	HKAS 112167^T^	2	China	MW989519	MW989495	MZ042642	NA	NA	Ren et al. (2021)
*H. krabiensis*	MFLUCC 16-0249^T^	2	Thailand	KX525742	KX525750	KX525758	KX525754	NA	Tibpromma et al. (2016)
*H. krabiensis* (*H. chiangmaiensis*)	MFLUCC 16-2817	2	Thailand	KY559394	NA	NA	NA	NA	Tibpromma et al. (2016)
*H. maharashtraense*	NFCCI 4879^T^	1	India	MZ099917	MZ147016	MZ130659	MZ130660	NA	Wijayawardene et al. (2021)
*H. maharashtraense*	NFCCI 4880	1	India	MZ147042	MZ147019	MZ130661	MZ130662	NA	Wijayawardene et al. (2021)
*H. megasporus*	CCF 5897	2	Panama	NA	LS398265	LS398419	LS398346	LS398444	Koukol et al. (2018)
*H. megasporus*	CCF 5898^T^	2	Panama	NA	LS398266	LS398420	NA	LS398445	Koukol et al. (2018)
*H. nabanheensis*	KUMCC 16-0149^T^	2	China	KY766059	KY766058	KY766061	NA	NA	Hyde et al. (2017)
*H. nujiangensis*	HKAS 144367	1	China	PV264903	PV264897	PV261935	PV261929	NA	Yang et al. (2025)
*H. nujiangensis*	HKAS 144368	1	China	PV264904	PV264898	PV261936	PV261930	NA	Yang et al. (2025)
***H. nyingchiensis***	**GZCC 25**-**0849^T^**	**2**	**China**	**PX413305**	**PX561052**	**PX436849**	**PX436845**	**PX436853**	**This study**
*H. pyriformis*	CGMCC 3.27462^T^	1	China	PP491962	PP491964	PP505452	PP505454	NA	Du et al. (2024)
*H. pyriformis*	UESTCC 23.0441	1	China	PP491963	PP491965	PP505453	PP505455	NA	Du et al. (2024)
*H. reticulatus*	CCF 5893	1	Panama	LS398267	LS398267	LS398421	LS398347	LS398446	Koukol et al. (2018)
*H. reticulatus* (*H. subiculosus*)	MFLUCC 15-0843	1	Thailand	KX259523	KX259521	KX259527	KX259529	NA	Hyde et al. (2016)
*H. sphaericoides*	CCF 5908^T^	1	Panama	LS398273	LS398273	LS398427	LS398352	LS398450	Koukol et al. (2018)
*H. sphaericoides*	CCF 5895	1	Panama	LS398270	LS398270	LS398424	LS398350	LS398447	Koukol et al. (2018)
*H. sphaericus*	PMA 116080	1	Panama	LS398281	LS398281	LS398431	LS398356	LS398454	Koukol et al. (2018)
*H. sphaericus*	PRC 4104	1	Panama	NA	LS398278	LS398430	LS398355	LS398453	Koukol et al. (2018)
*H. sphaericus*	MFLUCC 21-0036	1	Thailand	MW989516	MW989492	MZ042639	MZ042636	MZ042643	Ren et al. (2021)
*H. sphaericus* (*H. biconisporus*)	KUMCC 17-0183	2	China	MH260296	MH275063	MH412771	MH412755	NA	Tibpromma et al. (2018)
*H. sphaericus* (*H. chromolaenae*)	MFLUCC 16-2818	1	Thailand	KY559393	NA	NA	NA	NA	Tibpromma et al. (2017)
*H. sphaericus* (*H. pandanicola*)	MFLUCC 16-0251	2	Thailand	KX525743	KX525751	KX525759	KX525755	NA	Tibpromma et al. (2016)
*H. sphaericus* (*H. saikhuensis*)	MFLUCC 16-0266	1	Thailand	KX525740	KX525748	KX525756	KX525752	NA	Tibpromma et al. (2016)
*H. sphaericus* (*H. tectonae*)	MFLUCC 14-1141	2	Thailand	KU764696	KU144918	KU872758	NA	NA	Doilom et al. (2016)
*H. sphaericus* (*H. tectonae*)	MFLUCC 14-1140	2	Thailand	KU764695	KU144917	KU872757	KU712486	NA	Doilom et al. (2016)
*H. trangensis*	BCC 80742	1	Thailand	KY790601	KY790599	KY790607	KY790605	NA	Nuankaew et al. (2019)
*H. trangensis*	BCC 80741^T^	1	Thailand	KY790600	KY790598	KY790606	KY790604	NA	Nuankaew et al. (2019)
*H. tucumanensis*	CCF 5912	2	Panama	LS398288	LS398288	LS398435	LS398360	LS398458	Koukol et al. (2018)
*H. tucumanensis*	CCF 5915	2	Panama	LS398290	LS398290	LS398437	LS398362	NA	Koukol et al. (2018)
*H. turbinatus*	MFLUCC 21-0038^T^	2	Thailand	MW989518	MW989494	MZ042641	MZ042638	MZ042645	Ren et al. (2021)
*H. verrucosus*	CCF 5903^T^	1	Panama	LS398292	LS398292	LS398439	LS398364	LS398462	Koukol et al. (2018)
*H. verrucosus*	CCF 5892	1	Panama	LS398291	LS398291	LS398438	LS398363	LS398461	Koukol et al. (2018)
***H. xizangensis***	**GZCC 25-0850^T^**	**1**	**China**	**PX413303**	**PX561050**	**PX436847**	**PX436843**	**NA**	**This study**
***H. xizangensis***	**GZCC 25-0853**	**1**	**China**	**PX413304**	**PX561051**	**PX436848**	**PX436844**	**NA**	**This study**

**Table 2.** Strains and sequence accession numbers included for analysis of *Rhytidhysteron*. The newly generated sequences are in bold. “^T^” indicates the type strains. NA indicates sequence unavailability.

Fungal Species	Strain numbers	LSU	ITS	*tef*1-α	SSU
*Gloniopsis calami*	MFLUCC 15-0739	NG_059715	KX669036	KX671965	KX669034
*G. praelonga*	CBS 112415	FJ161173	NA	FJ161090	FJ161134
*Rhytidhysteron bannaense*	KUMCC 21-0483	OP526409	OP526399	OP572200	OP526395
*R. bannaense*	KUMCC 21-0482^T^	OP526408	OP526398	OP572199	OP526395
*R. bruguierae*	KUMCC 21-0484	OP482285	OP494090	OP572207	OP482277
*R. bruguierae*	MFLU 18-0571T	MN017833	NA	MN077056	MN017901
*R. bruguierae*	MFLUCC 17-1515	MN632452	MN632457	MN635661	MN632463
*R. bruguierae*	MFLUCC 17-1511	MN632454	MN632459	NA	MN632465
*R. bruguierae*	MFLUCC 17-1502	MN632453	MN632458	MN635662	MN632464
*R. bruguierae*	MFLUCC 17-1509	MN632455	MN632460	NA	MN632466
*R. camporesii*	KUMCC 21-0488	OP482286	OP494091	OP572208	OP482278
*R. camporesii*	HKAS 104277^T^	MN429072	MN429069	MN442087	NA
*R. chromolaenae*	MFLUCC 17-1516^T^	MN632456	MN632461	MN635663	MN632467
*R. coffeae*	KUMCC 21-0492	OP526406	OP605963	OP572201	OP526412
*R. coffeae*	KUMCC 21-0489^T^	OP526407	OP605964	OP572202	OP526413
*R. cozumelense*	A. Cobos-Villagrán951	MW939459	MZ056797	MZ457338	NA
*R. cozumelense*	T. Raymundo 7321	MW939460	MZ056798	MZ457339	NA
*R. erioi*	MFLU 16-0584^T^	MN429071	MN429068	MN442086	NA
*R. esperanzae*	T. Raymundo 6579	MZ477203	MZ056795	MZ457336	NA
*R. esperanzae*	R. Valenzuela 17206	MZ477204	MZ056796	MZ457337	NA
*R. hongheense*	KUMCC 21-0487	OP482287	OP494092	OP572209	OP482279
*R. hongheense*	KUMCC 20-0222	MW264193	MW264214	MW256815	MW264223
*R. hongheense*	HKAS112348	MW541820	MW541824	MW556132	MW541831
*R. hongheense*	HKAS112349	MW541821	MW541825	MW556133	MW541832
*R. hysterinum*	EB 0351	GU397350	NA	GU397340	NA
*R. hysterinum*	CBS 316.71	MH871912	MH860141	NA	NA
*R. ligustrum*	SICAUCC 20-0004^T^	MT062446	MT062850	MT075600	MT062451
*R. ligustrum*	SICAUCC 19-0007	MN956789	MN956777	MT027603	MN956798
*R. magnoliae*	KUMCC 21-0478	OP482288	OP494093	OP572210	OP482280
*R. magnoliae*	MFLUCC 18-0719^T^	MN989384	MN989383	MN997309	MN989382
*R. mangrovei*	MFLU 18-1894^T^	MK357777	MK425188	MK450030	NA
*R. mengziense*	KUMCC 21-0490^T^	OP526396	OP526402	OP572203	OP526414
*R. mengziense*	KUMCC 21-0491	OP526397	OP526403	OP572204	OP526415
*R. mesophilum*	A. Trejo 74	MW939461	MZ056799	MZ457340	NA
*R. mesophilum*	A. Cobos-Villagrán 1800	MW939462	MZ056800	MZ457341	NA
*R. mexicanum*	RV17107.1^T^	MT626028	MT626026	NA	NA
*R. mexicanum*	RV17107.2	MT626029	MT626027	NA	NA
*R. neorufulum*	KUMCC 21-0480	OP482290	OP494095	OP572212	OP482282
*R. neorufulum*	MFLUCC 21-0035	MZ346015	MZ346025	MZ356249	MZ346020
*R. neorufulum*	MFLUCC 13-0216^T^	KU377566	KU377561	KU510400	KU377571
*R. neorufulum*	GKM 361A	GQ221893	NA	NA	GU296192
*R. neorufulum*	HUEFS 192194	KF914915	NA	NA	NA
*R. neorufulum*	MFLUCC 12-0528	KJ418117	KJ418118	NA	KJ418119
*R. neorufulum*	CBS 306.38	FJ469672	NA	GU349031	GU296191
*R. neorufulum*	MFLUCC 12-0011	KJ418109	KJ206287	NA	KJ418110
*R. neorufulum*	MFLUCC 12-0567	KJ526126	KJ546124	NA	KJ546129
*R. neorufulum*	MFLUCC 12-0569	KJ526128	KJ546126	NA	KJ546131
*R. neorufulum*	EB 0381	GU397351	NA	NA	GU397366
*R. opuntiae*	GKM 1190	GQ221892	NA	GU397341	NA
*R. rufulum*	MFLUCC 14-0577^T^	KU377565	KU377560	KU510399	KU377570
*R. rufulum*	EB 0384	GU397354	NA	NA	GU397368
*R. rufulum*	EB 0382	GU397352	NA	NA	NA
*R. rufulum*	EB 0383	GU397353	NA	NA	GU397367
*R. rufulum*	MFLUCC 12-0013	KJ418111	KJ418112	NA	KJ418113
*R. sichuanense*	SICAUCC 19-0005^T^	MN956787	MN956775	MT027601	MN956796
*R. sichuanense*	SICAUCC 19-0006	MN956788	MN956776	MT027602	MN956797
*R. sichuanense*	SICAUCC 20-0005	MT062447	MT062851	MT075601	MT062452
*R. subrufulum*	SICAUCC 19-0011^T^	MN956793	MN956781	MT027607	MN956802
*R. subrufulum*	SICAUCC 19-0010	MN956792	MN956780	MT027606	MN956801
*R. subrufulum*	SICAUCC 20-0003	MT062445	MT062849	MT075599	MT062450
*R. tectonae*	KUMCC 21-0479	OP482291	OP494096	OP572213	OP482283
*R. tectonae*	MFLUCC 21-0037	MZ346013	MZ346023	MZ356247	MZ346018
*R. tectonae*	MFLUCC 21-0034	MZ346014	MZ346024	MZ356248	MZ346019
*R. tectonae*	MFLUCC 13-0710^T^	KU764698	KU144936	KU872760	KU712457
*R. thailandicum*	KUMCC 21-0493	OP482292	OP494097	OP572214	OP482284
*R. thailandicum*	MFLUCC 14-0503^T^	KU377564	KU377559	KU497490	KU377569
*R. thailandicum*	MFLUCC 12-0530	KJ526125	KJ546123	NA	KJ546128
*R. thailandicum*	MFLU17-0788	MT093472	MT093733	NA	MT093495
*R. thailandicum*	MFLUCC 13-0051	MN509434	MN509433	MN509435	NA
*R. xiaokongense*	KUMCC 20-0158	MZ346011	MZ346021	MZ356245	MZ346016
*R. xiaokongense*	KUMCC 20-0160^T^	MZ346012	NR_185637	MZ356246	NG_242421
***R. xizangense***	**GZCC 25-0851^T^**	**PX413306**	**PX561053**	**PX425314**	**PX413301**
***R. xizangense***	**GZCC 25-0852**	**PX413307**	**NA**	**PX436846**	**PX413302**
*R. yunnanense*	KUMCC 21-0485^T^	OP526404	OP526410	OP572205	OP526400
*R. yunnanense*	KUMCC 21-0486	OP526405	OP526411	OP572206	OP526401

## ﻿Taxonomy and results

### ﻿Phylogenetic analyses of *Hermatomyces*

**Figure 1.**
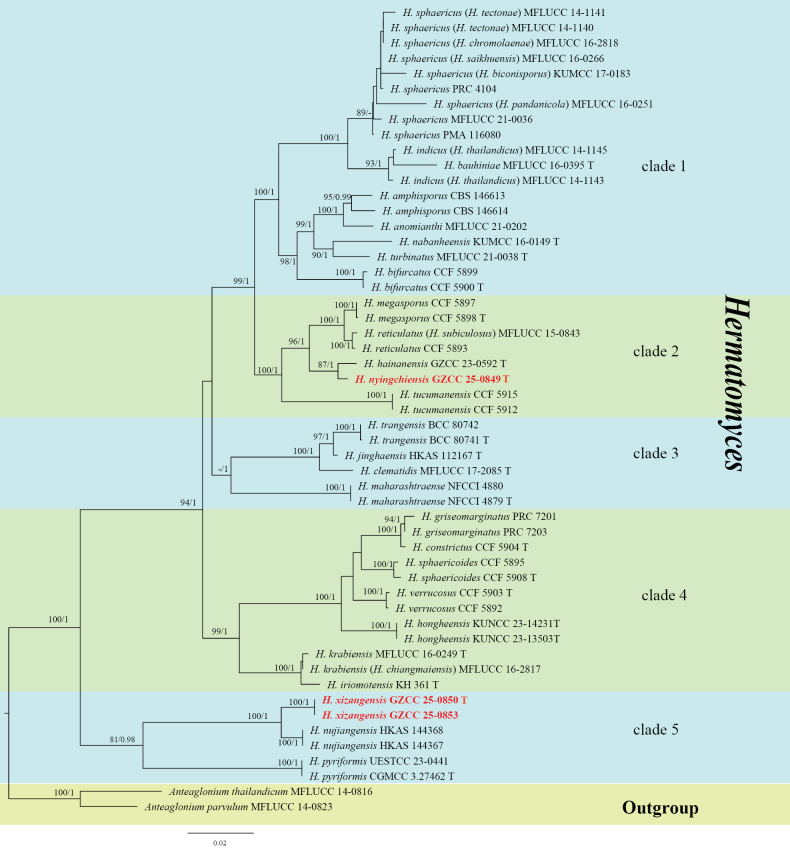
The ML tree based on a combined dataset of LSU, ITS, *tef*1-α, *rpb*2 and *tub*2 sequences. Bootstrap support values for ML greater than 75% and PP greater than 0.95 are marked near each node as ML/PP. Newly generated isolates are highlighted in bold red.

The concatenated dataset includes 53 strains, including the new collections. The best-scoring RAxML tree is shown in Fig. 1, with a final likelihood value of −15404.961825. The alignment contains a total of 3,809 characters (with gaps included), distributed as follows: ITS (1–503), LSU (504–1,320), *rpb*2 (1,321–2,325), *tef*1-α (2,326–3,249), and *tub*2 (3,250–3,809). The tree was rooted with *Anteaglonium parvulum* (MFLUCC 14-0823) and *A. thailandicum* (MFLUCC 14-0816). The alignment matrix exhibited 1,110 distinct patterns, with undetermined characters or gaps constituting 24.90% of the total dataset. Phylogenetic trees derived from ML and BI analyses exhibited fundamentally comparable topologies.

In the phylogenetic tree (Fig. 1), our strain of *Hermatomyces nyingchiensis* (GZCC 25-0849) formed a distinct clade that was sister to *H. hannanensis* (GZCC 23-0592) with 87% ML and 1 PP support. Our two strains of *Hermatomyces xizangensis* (GZCC 25-0850 and GZCC 25-0853) grouped together, forming a distinct clade sister to *H. nujiangensis* (HKAS 144367 and HKAS 144368) with 100% ML and 1 PP support.

#### ﻿ Hermatomyces nyingchiensis

Taxon classificationFungiFabalesFabaceae

G.L. Zhao, N.G. Liu & Y.Z. Lu sp. nov.

B9972C58-C34B-552F-A6E7-1A6F71857BC8

Fungal Names: FN 573085

Fig. 2

##### Etymology.

Referring to the location Nyingchi City, where the holotype was collected.

**Figure 2.**
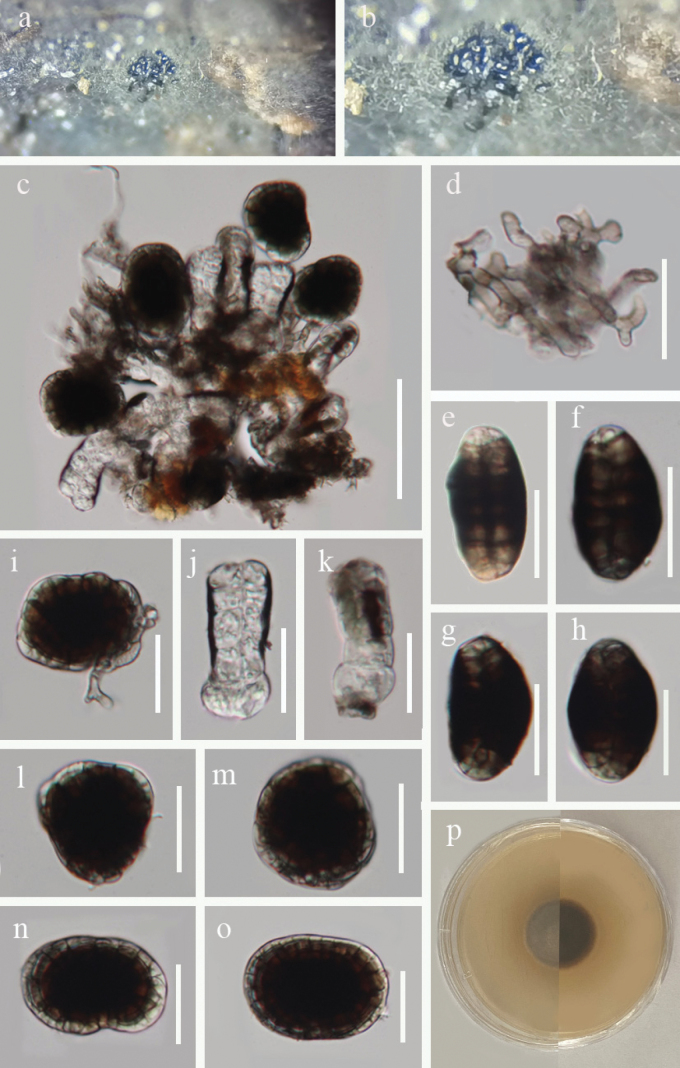
*Hermatomyces nyingchiensis* (HKAS 149901, holotype). **a, b.** Colonies on natural substrates; **c.** Conidial mass; **d.** Subicular hyphae; **e–h.** Lenticular conidia (side view); **i.** Conidiophore with conidium; **j, k.** Cylindrical conidia; **l–o.** Lenticular conidia (front view); **p.** Colony on PDA (left from above, right from below). Scale bars: 50 μm (**c**); 20 μm (**d–o**).

##### Holotype.

HKAS 149901.

##### Description.

***Saprobic*** on dead vine of *Pueraria* sp. in a terrestrial habitat. ***Sexual morph*** Undetermined. ***Asexual morph*** Hyphomycetous. *Colonies* on natural substrates superficial, effuse, scattered, comprising a greenish brown sterile mycelial outer region, and a dark brown to blackish brown center. ***Mycelium*** partly immersed and partly superficial, consisting of pale to brown, branched hyphae. ***Conidiophores*** micronematous, mononematous, geniculate, hyaline to pale brown, septate, thick-walled. ***Conidiogenous cells*** monoblastic, integrated, terminal, hyaline to pale brown. ***Conidia*** dimorphic, (1) cylindrical conidia 36–42 × 14–17 µm (x̄ = 38 × 15 µm, n = 15), hyaline to subhyaline, frequently exhibiting a distinct dark brown pigment, which either extends from the top downward or at the conidial rim, straight or broadly curved, phragmoseptate or muriform, with oblique septa occasionally present, constricted at septa, composed of two columns arising from one or two basal cells. (2) lenticular conidia 30–40 × 23–29 µm (x̄ = 35 × 27 µm, n = 30), 16–21 µm thick (x̄ = 18.5 µm, n = 10), in front view, subglobose to ellipsoidal, or slightly irregular, peripheral cells pale brown to brown, forming a distinct ring, constricted at the septa, central cells dark brown, in side view, rugby-shaped, oval, two distinct adpressed halves can be recognized, each half arranged by 6–10 cells, end cells subhyaline to pale brown, middle cells dark brown.

##### Culture characteristics.

Conidial germination was observed within 24 hours incubated on PDA medium at 28 °C. Colony reached a diameter of 1.9 cm after 19 days at room temperature, circular, dense, convex at the center, margin entire, pale brown on the front, dark brown in reverse.

##### Material examined.

China, Xizang Autonomous Region, Nyingchi City, Bomi County, (30°3'16"N, 95°12'20"E, 2320 m), on dead vine of *Pueraria* sp., 26 July 2024, G.L. Zhao, DDQGF14A (HKAS 149901, holotype), DDQGF14B (HKAS 149903, isotype) , ex-type culture GZCC 25-0849.

##### Notes.

*Hermatomyces nyingchiensis* resembles those *Hermatomyces* species that possess dimorphic conidia (Tibpromma et al. 2016; Hashimoto et al. 2017; Koukol et al. 2018; Ren et al. 2021; Zhang et al. 2023). In the phylogenetic tree (Fig. 1), our isolate (GZCC 25-0849) formed a distinct clade and was sister to *H. hainanensis* (GZCC 23-0592) with 87% ML-BS and 1 PP support (Fig. 1). However, the two conidial types of *H. nyingchiensis* are smaller than those of *H. hainanensis*: (1) cylindrical conidia, 36–42 × 14–17 µm vs. 51–67 × 16–24 μm; and (2) lenticular conidia, 30–40 × 23–29 µm vs. 44–52 × 29–39 µm (Zhang et al. 2023). Therefore, based on phylogenetic analyses and morphological characteristics, *H. nyingchiensis* is identified as a new species.

#### ﻿ Hermatomyces xizangensis

Taxon classificationFungiFabalesFabaceae

G.L. Zhao, N.G. Liu & Y.Z. Lu sp. nov.

399F781F-8D84-5A02-A30A-A7CD700C0FD9

Fungal Names: FN 573046

Fig. 3

##### Etymology.

Referring to the location Xizang Autonomous Region, where the fungus was collected.

**Figure 3.**
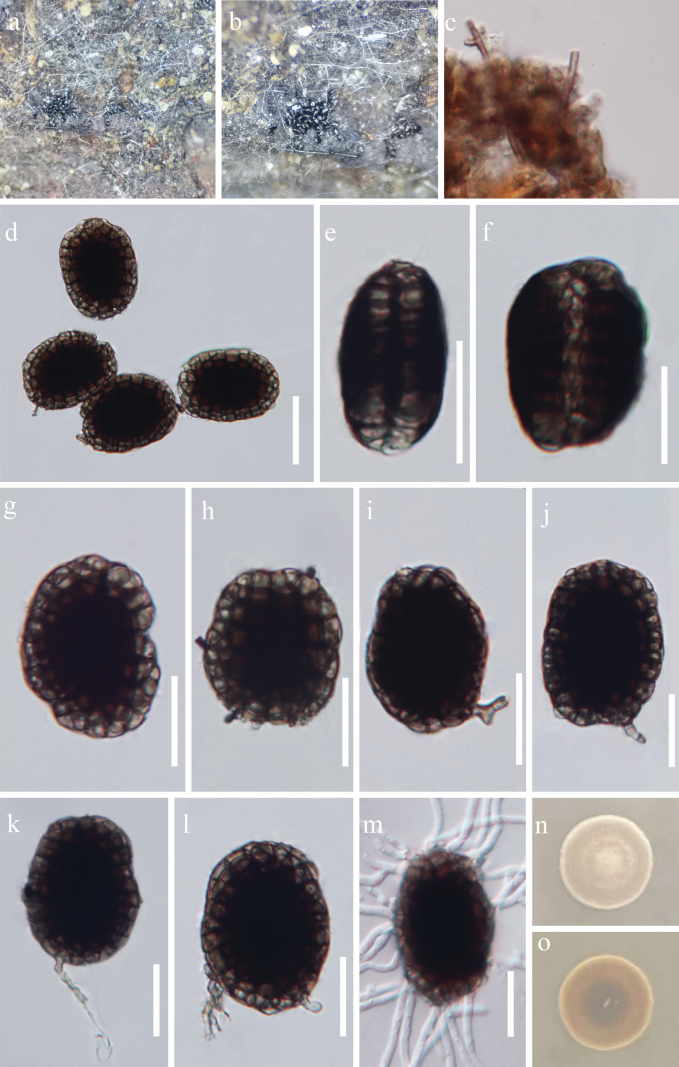
*Hermatomyces xizangensis* (HKAS 149902, holotype). **a, b.** Colonies on natural substrates; **c.** Subicular hyphae; **d–l.** Conidiophores and conidia; **m.** Germinated conidium; **n, o.** Colony on PDA (**n.** From above; **o.** From below). Scale bars: 50 μm (**d**); 20 μm (**e–m**).

##### Holotype.

HKAS 149902.

##### Description.

***Saprobic*** on dead vine of *Pueraria* sp. in a terrestrial habitat. ***Sexual morph*** Undetermined. ***Asexual morph*** Hyphomycetous. ***Colonies*** on natural substrate sporodochial, superficial, scattered, effuse, consisting of a velvety, sparse, brown sterile mycelial outer zone enclosing a brown to black, glistening, abundantly sporulating center, conidia not easily dislodged when disturbed. ***Mycelium*** mostly immersed and consisting of branched, smooth, septate, brown hyphae. ***Conidiophores*** micronematous, mononematous, curved, unbranched, light brown to brown thick-walled. ***Conidiogenous cells*** monoblastic, integrated, terminal, subcylindrical, hyaline to brown. ***Conidia*** 26–48 × 17–39 µm (x̄ = 36 × 27 µm, n = 30), 13–20 µm thick (x̄ = 15.5 µm, n = 15), monomorphic, lenticular, dry muriform, smooth, in front view, ellipsoidal, peripheral cells pale brown to brown, forming a distinct ring, constricted at the septa, central cells dark brown to blackish brown, in side view, rugby-shaped, oval, two distinct adpressed halves can be recognized, each half arranged by 8–10 cells, end cells subhyaline to pale brown, middle cells brown to dark brown.

##### Culture characteristics.

Conidia germinated within 24 hours on PDA medium at 28 °C. The colony reached 1.8 cm diam after 13 days at room temperature, circular, dense, bulge at middle with entire margin, reverse brown, with a lighter coloration at the margins and a darker pigmentation toward the center.

##### Material examined.

China, Xizang Autonomous Region, Nyingchi City, Bomi County, (30°3'16"N, 95°12'20"E, 2320 m), on dead vines of *Pueraria* sp., 26 July 2024, G.L. Zhao, DDQGF12A (HKAS 149902, holotype), ex-type culture GZCC 25-0850; *ibid*., DDQGF12B (HKAS 149904, isotype), ex-isotype culture GZCC 25-0853.

##### Notes.

Based on multi-gene phylogenetic analysis, our collections of *Hermatomyces xizangensis* (GZCC 25-0850 and GZCC 25-0853) formed an independent clade, sister to *H. nujiangensis* (HKAS 144367 and HKAS 144368) with 100% ML and 1 PP support (Fig. 1). Morphologically, conidia of *H. xizangensis* consist of 8–10 cells per half in side view, whereas those of *H. nujiangensis* have 15–22 cells (Yang et al. 2025). Furthermore, three *Hermatomyces* species, viz., *H. dimorphus*, *H. uniseriatus*, and *H. truncatus*, for which molecular data are lacking, are characterized by dimorphic conidia, including both lenticular and cylindrical types (Rao and de Hoog 1986; Leão-Ferreira et al. 2013; Koukol and Delgado 2019). In contrast, *H. xizangensis* produces only lenticular conidia. Therefore, based on molecular evidence and morphological comparisons, *H. xizangensis* is identified as a new species.

### ﻿Phylogenetic analyses of *Rhytidhysteron*

**Figure 4.**
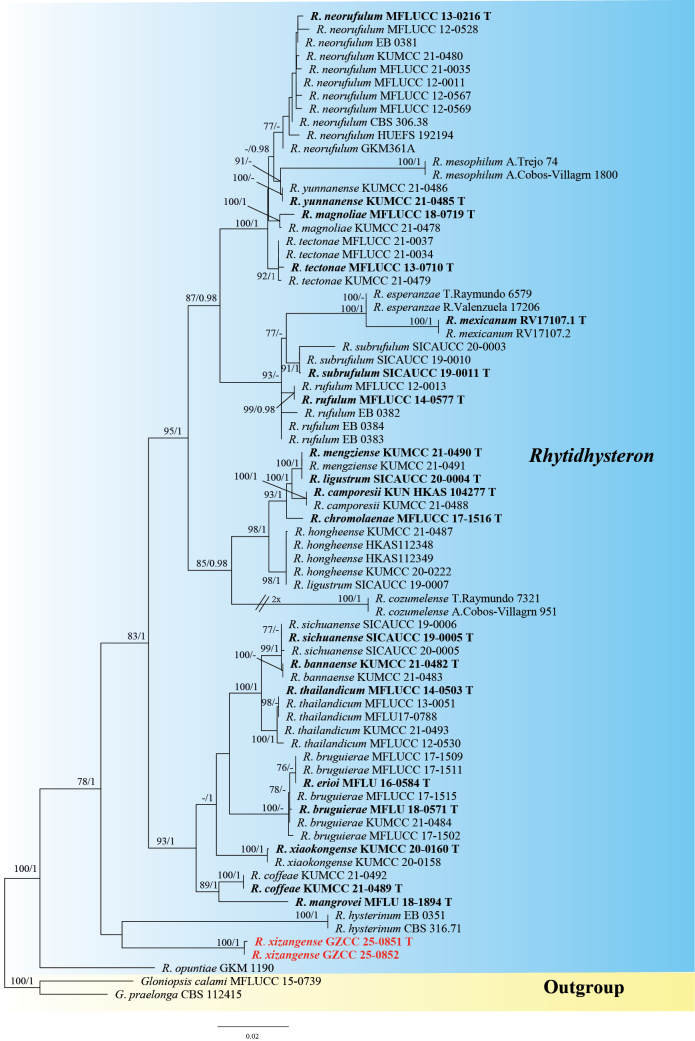
Phylogenetic tree from ML analysis based on the combined ITS, LSU, SSU, and *tef*1-α sequence datasets. Bootstrap support values for ML greater than 75% and PP greater than 0.95 are marked near each node as ML/PP. Newly generated isolates are highlighted in bold red.

The final ML tree of *Rhytidhysteron* exhibited a topological pattern congruent with that reported by Du et al. (2023). The tree was constructed using a concatenated dataset composed of four gene regions: ITS, LSU, SSU, and *tef*1-α, totaling 3,280 aligned characters (including gaps). The partitioning scheme was as follows: ITS (1–579), LSU (580–1,433), SSU (1,434–2,427), and *tef*1-α (2,428–3,280). *Gloniopsis calami* (MFLUCC 15-0739) and *G. praelonga* (CBS 112415) were selected as outgroup taxa. The ML analysis of the concatenated matrix yielded the best-scoring tree (Fig. 4) with a final optimization likelihood score of −11534.338749. The matrix had 831 distinct alignment patterns, and undetermined characters or gaps accounted for 20.42% of the total.

Within *Rhytidhysteron*, our new taxon, *R. xizangense* constitutes a distinct clade and is sister to *R. hysterinum* (EB 0351 and CBS 316.71).

#### ﻿ Rhytidhysteron xizangense

Taxon classificationFungiFabalesFabaceae

G.L. Zhao, N.G. Liu & Y.Z. Lu sp. nov.

B4CD6DCC-3A19-5648-845B-0340E8E466B9

Fungal Names: FN 573045

Figs 5, 6

##### Etymology.

Referring to the Xizang Autonomous Region, where the holotype was collected.

**Figure 5.**
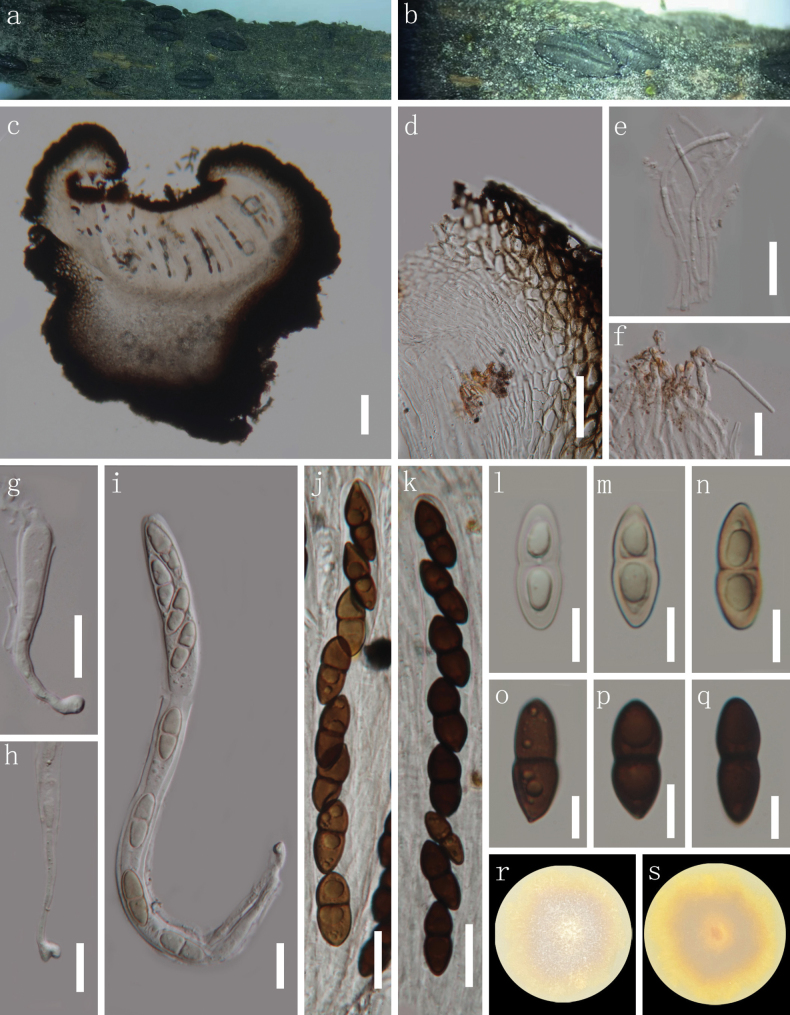
*Rhytidhysteron xizangense* (HKAS 149907, holotype). **a, b.** Appearance of hysterothecia on the host; **c.** Vertical section through hysterothecium; **d.** Exciple; **e.** Pseudoparaphyses; **f.** Epithecium mounted in water; **g, h.** Pedicel of asci; **i–k.** Asci; **l–q.** Ascospores; **r, s.** Colony on PDA medium. Scale bars: 100 μm (**c**); 50 μm (**d**); 20 μm (**e–k**); 10 μm (**l–q**).

##### Holotype.

HKAS 149907.

**Figure 6.**
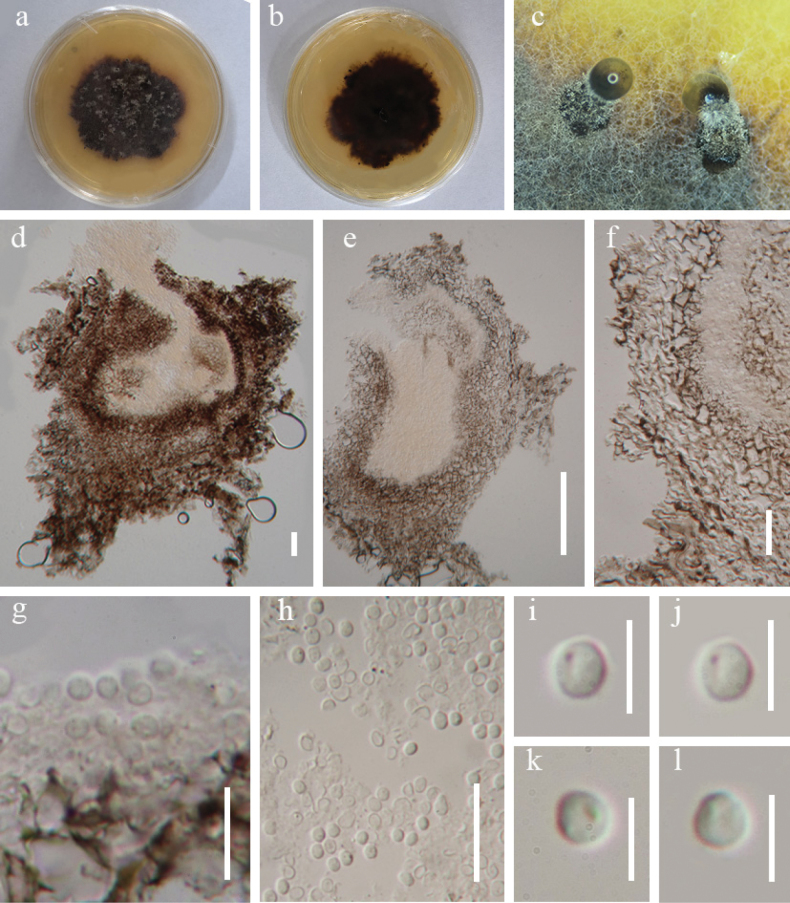
*Rhytidhysteron xizangense* (HKAS 149907, holotype) on PDA. **a, b.** Obverse and reverse colony on PDA; **c.** Conidiomata on PDA; **d, e.** Vertical section through conidiomata; **f, h.** Conidiomatal wall; **g, h.** Conidiogenous cells and conidia; **i–l.** Conidia. Scale bars: 100 μm (**d, e**); 20 μm (**f, h**); 10 μm (**g**); 5 μm (**i–l**).

##### Description.

***Saprobic*** on dead vine of *Pueraria* sp. in a terrestrial habitat. **Sexual morph *Ascomata*** 1432–1818 µm long × 780–1265 µm wide × 648–1100 µm high (x̄ = 1622 × 959 × 822 µm, n = 5), scattered to gregarious, semi-immersed to superficial, hysterothecial, bilabiate, closed, black, perpendicular striae. ***Exciple*** 150–220 µm (x̄ = 170 µm, n = 5) wide, consisting of dark brown, thick-walled cells arranged in a *textura angularis* structure, outer layer brown to dark brown, inner layer hyaline. ***Hamathecium*** 2–3 μm (x̄ = 2.5, n = 10) wide at the base, 3.5–6.5 μm (x̄ = 4.5, n = 10) wide at swollen tips, dense, brown at the swollen apex, hyaline at the base, septate, branched, pseudoparaphyses, forming a brown epithecium above the asci, swollen with dense septa at the apex. ***Asci*** 199–275 × 13–18 µm (x̄ = 224 × 16 µm, n = 20), 8-spored, bitunicate, clavate to cylindrical, short pedicel, rounded at the apex, with an ocular chamber. ***Ascospores*** 23–30 × 9–14 µm (x̄ = 26.5 × 11 µm, n = 30), uni-seriate, slightly overlapping, 1-septate, hyaline when immature, brown to dark brown when mature, fusiform to ellipsoidal, slightly pointed at both ends, thick-walled. ***Asexual morph*** Sporulation in PDA media, coelomycetous. ***Conidiomata*** 260–519 µm high × 391–467 µm wide (x̄ = 411 × 424 µm, n = 10), superficial, subglobose, solitary or aggregated, dark brown to black, thick-walled. ***Conidiomata wall*** 63–288 µm (x̄ = 124 µm, n = 15) wide, consisting of brown, thick-walled cells arranged in a *textura angularis* structure. ***Conidia*** 2.5–4.5 µm diam. (x̄ = 3.0 µm, n = 40), globose, hyaline, smooth-walled.

##### Culture characteristics.

Conidia germinated within 24 hours on PDA medium at 28 °C. After one month of incubation at room temperature, colonies reached a diameter of 3.35 cm. Colonies velvety, subrounded, with wavy margins, yellow in the center, brown in the middle, with light yellow at the edge.

##### Material examined.

China, Xizang Autonomous Region, Nyingchi City, Bomi County (29°53'11"N, 95°42'9"E, 2720 m), on dead vine of *Pueraria* sp., 26 July 2024, G.L. Zhao, CGGF7A (holotype, HKAS 149907), ex-type living culture GZCC 25-0851; *ibid*., CGGF7B (isotype, HKAS 149908), ex-isotype living culture GZCC 25-0852.

##### Notes.

*Rhytidhysteron xizangense* is similar to *R. neohysterinum* in the shapes of ascomata, asci, and ascospores (Cobos-Villagrán et al. 2020). However, in the phylogenetic tree (Fig. 4), *R. xizangense* (GZCC 25-0851 and GZCC 25-0852) formed an independent clade and is sister to *R. hysterinum* (EB 0351 and CBS 316.71). BLAST analyses of the ITS and LSU sequences revealed that our strain (GZCC 25-0851) shares 92% (36/439, including 8 gaps) and 97% (21/786, including 0 gap) similarity with *R. hysterinum* (CBS 316.71), respectively. Morphologically, *Rhytidhysteron xizangense* differs from *R. hysterinum* by its larger ascomata (780–1265 µm vs. 500 µm) (Thambugala et al. 2016; Ren et al. 2022; Du et al. 2023). Therefore, based on molecular data and morphological comparisons, we introduce *Rhytidhysteron xizangense* as a new species.

## ﻿Discussion

In this study, three novel species, namely *Hermatomyces nyingchiensis*, *H. xizangensis*, and *Rhytidhysteron xizangense*, are introduced. These collections were isolated from decaying vines of *Pueraria* sp. in the Xizang Autonomous Region. The findings of this study further clarify the species diversity within *Hermatomyces* and *Rhytidhysteron* while also supplementing the distribution information of fungi associated with *Pueraria* hosts.

Among the 26 recognized species of *Hermatomyces* (Table 1), 15 species exhibit conidial dimorphism, such as *H. amphisporus*, *H. bifurcatus*, *H. hainanensis*, *H. nabanheensis*, and *H. turbinatus* (Hyde et al. 2017; Koukol et al. 2018; Delgado et al. 2020; Ren et al. 2021; Zhang et al. 2023). Nine species produce exclusively monomorphic conidia, viz., *H. griseomarginatus*, *H. hongheensis*, *H. maharashtraense*, *H. pyriformis*, *H. reticulatus*, *H. sphaericoides*, *H. trangensis*, *H. verrucosus*, and *H. xizangensis* (Koukol et al. 2018, 2025; Nuankaew et al. 2019; Wijayawardene et al. 2021; Du et al. 2024; Shen et al. 2024). One species, *H. sphaericus*, is known to exhibit either monomorphic or dimorphic conidia (Doilom et al. 2016; Tibpromma et al. 2016, 2017, 2018; Koukol et al. 2018; Ren et al. 2021), whereas *H. anomianthi* is known solely from its sexual morph (de Silva et al. 2022). Phylogenetic analysis indicates that conidial morphology (monomorphic vs. dimorphic) does not exhibit a clear phylogenetic clustering pattern. For instance, *H. megasporus* (dimorphic conidia) and *H. reticulatus* (monomorphic conidia) cluster in clade 2 (Fig. 1) (Hyde et al. 2016; Koukol et al. 2018), while *H. jinghaensis* (dimorphic conidia) and *H. trangensis* (monomorphic conidia) also group together in clade 3 (Fig. 1) (Nuankaew et al. 2019; Ren et al. 2021). Furthermore, conidial type shows no significant correlation with biogeographical distribution. Although both *H. hongheensis* and *H. nabanheensis* were collected from Yunnan, China, the former produces monomorphic conidia, whereas the latter develops dimorphic conidia (Hyde et al. 2017; Shen et al. 2024). To elucidate the underlying mechanisms governing the formation of dimorphic conidia and monomorphic conidia in *Hermatomyces*, broader sampling is essential across diverse geographical regions, ecological niches, and developmental stages. Future research integrating comparative genomics, transcriptomics, and ecological niche modeling will be crucial for elucidating the evolutionary and ecological drivers of conidial dimorphism within the genus.

## Supplementary Material

XML Treatment for Hermatomyces nyingchiensis

XML Treatment for Hermatomyces xizangensis

XML Treatment for Rhytidhysteron xizangense

## ﻿Additional information

### Use of AI

No use of AI was reported.

### Author ORCIDs

Gui-Li Zhao https://orcid.org/0000-0001-8370-4844

Yong-Zhong Lu https://orcid.org/0000-0002-1033-5782

Xing-Juan Xiao https://orcid.org/0009-0003-8769-4534

Ying Liu https://orcid.org/0009-0003-4850-4929

Qiang Chen https://orcid.org/0009-0004-2035-6562

Ning-Guo Liu https://orcid.org/0000-0002-9169-2350

### Data availability

All of the data that support the findings of this study are available in the main text.
